# Simulation Study of the Localization of a Near-Surface Crack Using an Air-Coupled Ultrasonic Sensor Array

**DOI:** 10.3390/s17040930

**Published:** 2017-04-22

**Authors:** Steven Delrue, Vladislav Aleshin, Mikael Sørensen, Lieven De Lathauwer

**Affiliations:** 1 Wave Propagation and Signal Processing Research Group, KU Leuven Kulak, 8500 Kortrijk, Belgium; 2 Joint International Laboratory LICS/LEMAC, Institute of Electronics, Microelectronics and Nanotechnologies, UMR CNRS 8520, 59 652 Villeneuve d’Ascq CEDEX, France; Vladislav.Aleshin@iemn.univ-lille1.fr; 3 Group Science, Engineering and Technology, KU Leuven Kulak, 8500 Kortrijk, Belgium; mikael.sorensen@kuleuven.be (M.S.); lieven.delathauwer@kuleuven.be (L.D.L.); 4 Department of Electrical Engineering (ESAT), STADIUS, KU Leuven, 3001 Leuven-Heverlee, Belgium

**Keywords:** non-destructive testing, Nonlinear Air-Coupled Emission (NACE), crack localization, Direction Of Arrival (DOA), ultrasonic sensor array

## Abstract

The importance of Non-Destructive Testing (NDT) to check the integrity of materials in different fields of industry has increased significantly in recent years. Actually, industry demands NDT methods that allow fast (preferably non-contact) detection and localization of early-stage defects with easy-to-interpret results, so that even a non-expert field worker can carry out the testing. The main challenge is to combine as many of these requirements into one single technique. The concept of acoustic cameras, developed for low frequency NDT, meets most of the above-mentioned requirements. These cameras make use of an array of microphones to visualize noise sources by estimating the Direction Of Arrival (DOA) of the impinging sound waves. Until now, however, because of limitations in the frequency range and the lack of integrated nonlinear post-processing, acoustic camera systems have never been used for the localization of incipient damage. The goal of the current paper is to numerically investigate the capabilities of locating incipient damage by measuring the nonlinear airborne emission of the defect using a non-contact ultrasonic sensor array. We will consider a simple case of a sample with a single near-surface crack and prove that after efficient excitation of the defect sample, the nonlinear defect responses can be detected by a uniform linear sensor array. These responses are then used to determine the location of the defect by means of three different DOA algorithms. The results obtained in this study can be considered as a first step towards the development of a nonlinear ultrasonic camera system, comprising the ultrasonic sensor array as the hardware and nonlinear post-processing and source localization software.

## 1. Introduction

Driven by an ever-increasing demand of the consumer market for better quality products and durable solutions, new and advanced materials are being developed and employed in various application fields. However, despite the high quality of these products, tiny manufacturing faults and/or mechanical or thermal loading may induce defects such as cracks and delaminations, which are often invisible to the naked eye. Such hidden damage in structures can gradually develop into larger flaws under cyclic loading, endangering the integrity of the whole structure and leading to catastrophic failure if the damage remains undetected. In order to prevent this, effective Non-Destructive Testing and Evaluation (NDT&E) methods are being developed that enable the integrity of structures to be assessed, both at the production line or at regular time intervals while already in service. To reduce operational costs and improve reliability and performance, a common goal of researchers, designers and manufacturers is to develop a real-time non-destructive inspection system, capable of detecting incipient damage and allowing an easy interpretation of the results.

Among many other NDT techniques, ultrasonic testing is definitely one of the most used methods. In general, ultrasonic techniques rely on the partial reflection and transmission of ultrasonic waves at the interface between two different materials, or at heterogeneities (linear techniques), or in the nonlinear regime, on the investigation of the amplitude dependence of material parameters (e.g., power law strain dependence of the moduli, velocities, attenuation), which can for instance be evidenced by modifications in the spectral content and lack of scalability with amplitude (Nonlinear Elastic Wave Spectroscopy (NEWS) techniques). Compared to the linear methods, NEWS techniques have proven to be extremely sensitive to early stage damage evaluation in materials [1,2,3,4,5]. In the case of cracks and delaminations, the use of finite amplitude (or nonlinear) ultrasonic waves may be beneficial to overcome the defect’s specific activation threshold below which the defect remains permanently closed, thereby enforcing a repetitive opening and closing of its interfaces. The resulting local contact behavior (commonly referred to as kissing, breathing or clapping) gives rise to a nonlinear stress-strain response at the defect location, which is inherently different in tension than in compression (violation of the simple Hooke’s law). This is evidenced by a broadening of the frequency spectrum of the nonlinear structural response, which is no longer dominated only by the frequency of the excitation signal. Higher order harmonics and subharmonics, whose frequencies have a prescribed relation with the excitation frequency, appear in the spectrum as a result of the nonlinearity [2,3,6,7,8]. In time domain analysis, the evidence of defects is merely demonstrated by a lack of scalability of the response signals with amplitude [9] and an asymmetry of the recorded signals with respect to the polarity of the excitation [10].

In many practical applications employing finite amplitude waves for damage diagnostics, the sample under investigation is excited by a source (e.g., piezoelectric sensor), which stimulates and triggers a nonlinear response from the material microstructure at the defect position. The resulting nonlinear symptoms, such as harmonics and combination frequencies, propagate throughout the sample and are subsequently recorded and analyzed by means of one or multiple sensors glued to the sample. This information can finally be used for defect detection and characterization [4,5,11]. In the case that the defect also needs to be localized, Time Reversal (TR) imaging [12,13] or Sparse Array Tomography (SAT) [14,15,16] can be used. Both techniques have already proven their efficiency and reliability in many numerical and experimental studies. However, one major drawback associated with these techniques is that they both require a significant number of sensors to be connected to the test sample, which is not always desirable in industrial applications, especially when large or delicate structures need to be monitored. To overcome the limitations of traditional sensor coupling for defect localization, non-contact ultrasonic techniques can be used [17,18]. Over the last few decades, several non-contact-based NDT techniques have been developed and used. However, until now, only a few of these techniques were developed for nonlinear ultrasonic applications. Ouchi et al. [19], for instance, proposed an imaging method using a subharmonic phased array for crack evaluation using surface acoustics waves with water immersion. The described technique allows non-contact, accurate measurements of closed crack length and imaging of the distribution of open and closed parts of the crack. Even though this technique has proven to be very promising, an important drawback of this method is that it cannot be used for all types of materials, as some of them can be damaged when using liquid couplants. Therefore, other approaches use air-coupled methods, where the surface vibration of bulk waves, guided waves or standing waves in response to piezoelectric excitation is for instance analyzed by a scanning laser Doppler vibrometer, and defects can be localized and imaged by filtering out the higher harmonics from the recorded normal surface displacements of the structure [8,20]. Despite the promising experimental results using Scanning Laser Vibrometry (SLV) in recent years, this technique has the disadvantage that it is very sensitive to small variations in the sample’s surface-to-beam inclination, making it difficult to scan optically-rough targets. Moreover, as SLV operates in a scanning mode, it may be exceedingly time consuming, depending on the desired resolution and size of the object under investigation. A potentially alternative approach to locate and visualize defects in nonlinear NDT consists of measuring their nonlinear airborne emission. As recently demonstrated, both experimentally and numerically, the nonlinear vibrations generated by clapping contacts cause high-frequency ultrasonic radiation into the ambient air, referred to as Nonlinear Air-Coupled Emission (NACE) [20,21]. Once excited, defects thus behave as localized sources of nonlinear emission, broadcasting harmonics from the respective defect locations. Unlike SLV, which analyzes the laser light that is locally reflected from the specimen, NACE imaging analyzes the direct nonlinear acoustic radiation by the defects. Recent experiments showed that defect detection using NACE was possible, even in the case of rough surfaces, using a weakly-focused (cm range depth of focus), high-frequency, air-coupled receiver [20,22]. Defect localization, however, also requires NACE-imaging to be operated in scanning mode, similar to SLV.

The goal of the current paper is to avoid the time-consuming scanning operation that is inherent to both SLV and NACE imaging, by detecting the nonlinear airborne components in all directions using an air-coupled ultrasonic sensor array. An efficient post-processing step using appropriate localization algorithms enables rapidly and accurately inferring the location of the defect. The proposed configuration is similar to the standard microphone arrays used in acoustic cameras that are frequently being used for localization and identification of noise sources [23,24]. However, since the currently available acoustic camera systems operate in a relatively low frequency range (from a few 100 Hz up to 10 kHz, with some models capable of reaching 20 kHz) and have a nominal spatial resolution of a few cm, they have never been used for localization of incipient damage (i.e., mm to sub-mm sizes). Apart from the frequency range and the focus on detecting damage instead of noise, the methodology of the ultrasonic receiver array is different as well from the current acoustic camera systems. In order to enable the localization of early stage damage, the defect’s nonlinear reaction in response to an external ultrasonic excitation needs to be triggered and filtered out of the direct recorded airborne signals. The nonlinear filtering techniques required to achieve this are also not included in traditional acoustic camera systems.

The research conducted in this paper focuses on the theoretical study and numerical simulation of an air-coupled ultrasonic sensor array that can be applied for fast and reliable, in-line detection and localization of incipient (nonlinear) damage features, such as delaminations and micro-cracks. The paper starts in Section 2 with a detailed description of the forward model that combines a 2D time domain model for nonlinear Lamb wave propagation in a cracked sample, with a spectral solution for the nonlinear air-coupled emission. The results obtained using this model clearly illustrate the generation of nonlinear features by a near-surface crack and the resulting leakage of nonlinear waves from the defect sample into the ambient air. In Section 3, we will investigate the capabilities of locating the near-surface crack by measuring the nonlinear airborne emission of the defect using a uniform linear air-coupled ultrasonic sensor array. A comparison between three different localization algorithms will be carried out in order to explore the pros and cons of each method with respect to their use in an ultrasonic camera system.

## 2. Forward Model: Generation and Emission of Nonlinear Features

The fundamental concept of the proposed sensor system is based on the detection of nonlinear waves leaking from the defect sample into the ambient air (i.e., NACE). The sensor elements will detect the NACE signals and use them to identify the defect location. In order to demonstrate this new principle, we first need to model the concept of NACE. This is done by implementing a two-step 2D numerical model in the finite element-based commercially available software package COMSOL Multiphysics^®^. The first step of the model contains a time domain model for the calculation of nonlinear Lamb wave propagation in an aluminum sample with a horizontally-oriented near-surface crack. In the second step, NACE radiation patterns are calculated by bridging the results of the time domain solution to a spectral solution of air. A similar modeling approach was already used for studying the influence of different defect parameters on NACE radiation patterns [21]. In this section, we will briefly discuss the implementation of this two-step procedure.

### 2.1. Generation of Nonlinearities at a Near-Surface Crack

For the simulation of nonlinear wave-crack interactions, we developed a numerical tool consisting of two components: (1) a solid mechanics module that solves the elastic wave propagation problem and (2) an external custom-developed contact model that takes into account the physics of normal and tangential contact interactions between the crack faces and that is based on the following essential features:

- the considered contact model includes friction based on the Coulomb friction law;
- the internal contact/crack surfaces have a nontrivial topography (e.g., roughness);
- the normal load-displacement dependency for rough surfaces requires some information on roughness statistics, or otherwise, it can be measured directly for an engineered contact;
- the tangential interactions appear during shift; rolling and torsion as movement types are not considered;
- plasticity and adhesion are neglected.

The contact model is integrated in the solid mechanics module, as it provides the necessary boundary conditions, represented as a link between contact loads and displacements, to be imposed at the internal crack boundaries in the solid mechanics unit. The approach to calculate the desired physics-based relationships between normal and tangential loads *N* and *T* (i.e., forces per unit of nominal contact area) and normal and tangential displacements *a* and *b* requires the introduction of an intermediate scale, or in other words, a mesoscopic cell. On the one hand, the size of this cell is considerably less than both the crack size and the wavelength, so that the calculated macroscopic elastic fields are approximately uniform within each cell. On the other hand, the cell size is much greater than the scale of roughness. The contact in a mesoscopic cell can evolve in one of three regimes: contact loss (N=T=0), total sliding (|T|=μN, with μ the friction coefficient) and partial slip (|T|<μN). The latter occurs when both stick and slip areas are present in the contact zone and is only possible due to the presence of surface roughness. Indeed, in the case of perfectly smooth surfaces, the condition |T|<μN actually corresponds to the state of stick in accordance to Coulomb’s law of friction. In the partial slip regime, the required relation between loads and displacements is obtained via the Method of Memory Diagrams (MMD) [25]. MMD is based on the use of a memory diagram function that contains all memory information present in the system. This memory diagram function evolves in accordance to a number of prescribed rules and offers the possibility to calculate hysteretic tangential reaction curves as a function of displacement histories a(t) and b(t). However, to do so, MMD requires the knowledge of the normal contact reaction N=N(a) for the system under study. Based on some theoretical [25] and experimental [26] arguments, the normal reaction curve N(a) is considered to have a quadratic dependency:

(1) $$N\left(a\right)=C^{2}a^{2},\;\left(a\geq 0\right),$$

where $C=6\times 10^{10}$ Pa${}^{1/2}$m${}^{-1}$. This approximation only works for small *a*, which corresponds to weak acoustic strains.

In order to obtain the solution to the full mesoscopic contact problem and derive the load-displacement relationship in all three regimes, for any arbitrary combination of displacements and their histories, the following approach is followed. First, the tangential displacement *b* is presented as a sum of two components:

(2) $$b=b_{0}+\tilde{b},$$

where $b_{0}$ corresponds to the displacement achieved in the total sliding regime and $\tilde{b}$ is a component that reflects partial slip and the ability of asperities to recede under tangential load. Then, a solution for each of the three contact regimes can be defined:

- Contact loss occurs when a≤0. In this case, no contact interaction is present, meaning that N=T=0. As a result, asperities remain unstrained at this moment, meaning that $\tilde{b}=0$, and hence, $b_{0}=b$. These modifications will guarantee correct evolution of the memory diagram function once the crack faces get in contact.
- Partial slip occurs when a>0 and $|\tilde{b}|<\theta \mu a$, with θ a material constant depending on Poisson’s ratio:
  (3) $$\theta =\frac{2-\nu }{2(1-\nu )}.$$
  The second identification criterion actually corresponds to Coulomb’s condition for stick regimes, which, in this case, is written for displacements instead of the more traditional condition written for forces. In the partial slip case, the total sliding contribution $b_{0}$ remains unchanged, and hence, $\tilde{b}=b-b_{0}$. Using this new value for $\tilde{b}$ as an argument in the MMD algorithm, the tangential load *T* can be calculated. The magnitude of the normal load *N* is calculated using Equation (1).
- Total sliding occurs when a>0 and $|\tilde{b}|\geq \theta \mu a$. Similar to the partial slip case, the second identification criterion corresponds to Coulomb’s condition for slip regimes, again written for displacements. In this case, the tangential load is determined in accordance with the Coulomb friction law, T=±μN, where the magnitude of *N* is again calculated using Equation (1). To guarantee correct evolution of the memory diagram function during the next time steps, we also set $\tilde{b}=\pm \theta \mu a$, as this is the maximum possible tangential displacement corresponding to elastic deformation of asperities, and, as a result, $b_{0}=b-\tilde{b}$.

Finally, the calculated link between contact loads and displacements is used as an internal boundary condition defined at the crack boundaries in the solid mechanics unit. In COMSOL Multiphysics^®^, this is done by using the ‘thin elastic layer’ boundary condition. For a more detailed description of the theoretical contact model and its numerical implementation, we refer to [27,28].

**Figure 1:**
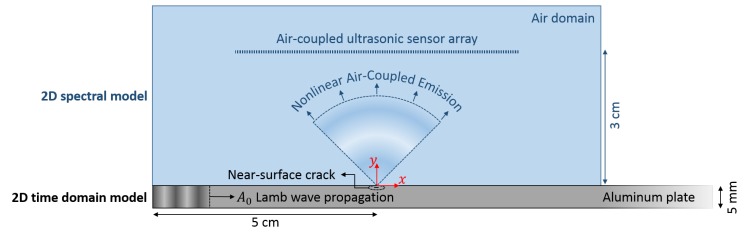
Illustration of the model geometry consisting of a 5-mm aluminum plate with a horizontally-oriented near-surface crack of 1 mm in length and positioned at a depth of 0.2 mm. An $A_{0}$ Lamb mode is excited at the leftmost boundary and propagates through the sample. While interacting with the crack, nonlinearities are being generated, causing high-frequency ultrasonic radiation in the ambient air (i.e., nonlinear air-coupled emission). The nonlinear radiation is captured by an air-coupled ultrasonic sensor array to be used for defect localization. The sensor array is positioned 3 cm above the sample.

The numerical tool for wave-crack interaction is now illustrated for the case of a near-surface crack in an aluminum plate. The aluminum sample has a density ρ=2700 kg/m${}^{3}$, Young’s modulus E=70 GPa and Poisson’s ratio ν=0.33. The plate has a thickness of 5 mm and a length of 35 cm. A horizontally-oriented near-surface crack with a length of 1 mm is introduced in the sample at a depth of 0.2 mm. The center position of the crack is located at a distance of 5 cm from the left boundary of the sample, as illustrated in Figure 1. The aluminum plate is excited by applying a prescribed sinusoidal displacement at a frequency of 100 kHz across the entire left boundary of the plate. The amplitude of the sinusoidal displacements depends on the depth of the sample and corresponds to the theoretically-calculated $A_{0}$ Lamb displacement profiles in *x*- and *y*-direction. On the rightmost boundary of the plate, a low-reflecting boundary condition was imposed to minimize the presence of unwanted reflections from the edges of the computational region. Doing so, the modeled geometry actually represents a plate that is infinitely long in the *x*-direction. The top and bottom boundaries of the plate were set to be free boundaries. At the internal crack surfaces, a thin elastic layer boundary condition is defined, according to the above description.

The (nonlinear) wave propagation problem is solved using the implicit generalized alpha time-dependent solver, which is the preferred solver to be used for structural mechanics problems in COMSOL^®^. In order to get accurate solutions, the time step Δt is set equal to 50 ns, corresponding to 200 time steps per wave cycle. The total duration *T* of the simulation is 200 μs, corresponding to 20 periods of the excited Lamb wave at 100 kHz. The aluminum domain is meshed using quadratic triangular elements with a maximum element size of 1.7 mm, ensuring convergence for at least the excited wave and the generated second harmonic wave. Smaller mesh elements are generated in the region of the crack, since the MMD algorithm requires a small spatial discretization size in order to obtain stable and accurate solutions. Here, a fixed number of 20 mesh elements at the internal crack boundary is adopted, corresponding to an element size of 0.05 mm.

**Figure 2:**
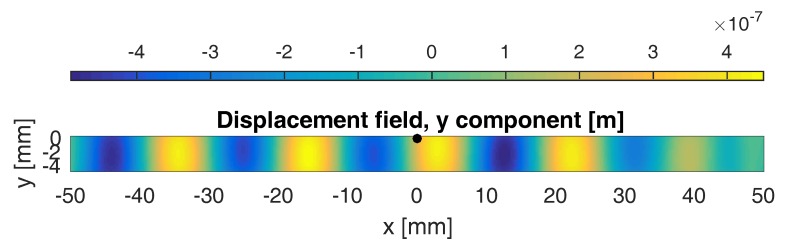
Snapshot of the calculated *y*-component of the displacement field in the aluminum sample, clearly illustrating the presence of an $A_{0}$ guided Lamb wave. The black dot indicates the location of the near-surface crack.

**Figure 3:**
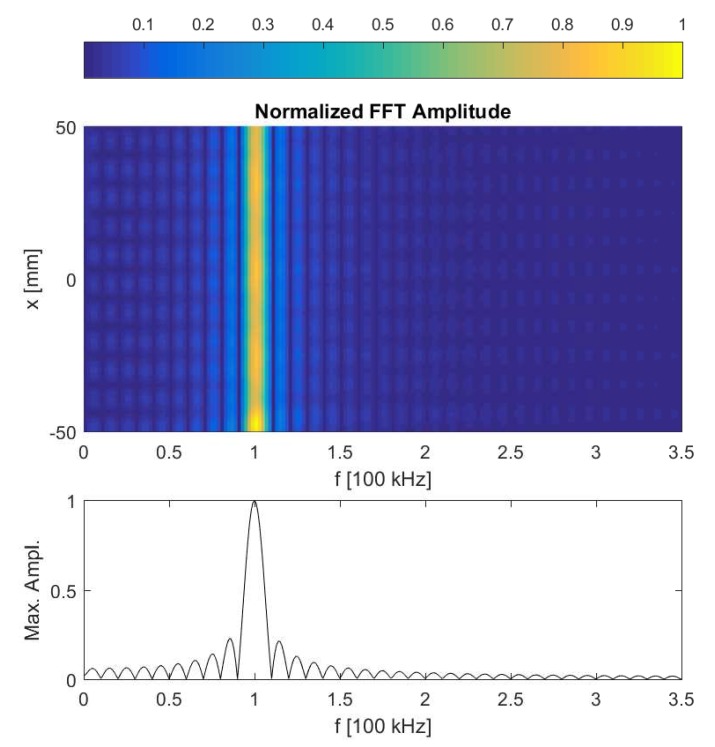
(Top) Frequency spectra of the calculated normal displacement signals for a number of points on the top surface of the plate, with *x*-coordinates ranging from −50 mm to 50 mm. (Bottom) Normalized maximum FFT amplitude response measured along the top surface of the plate.

**Figure 4:**
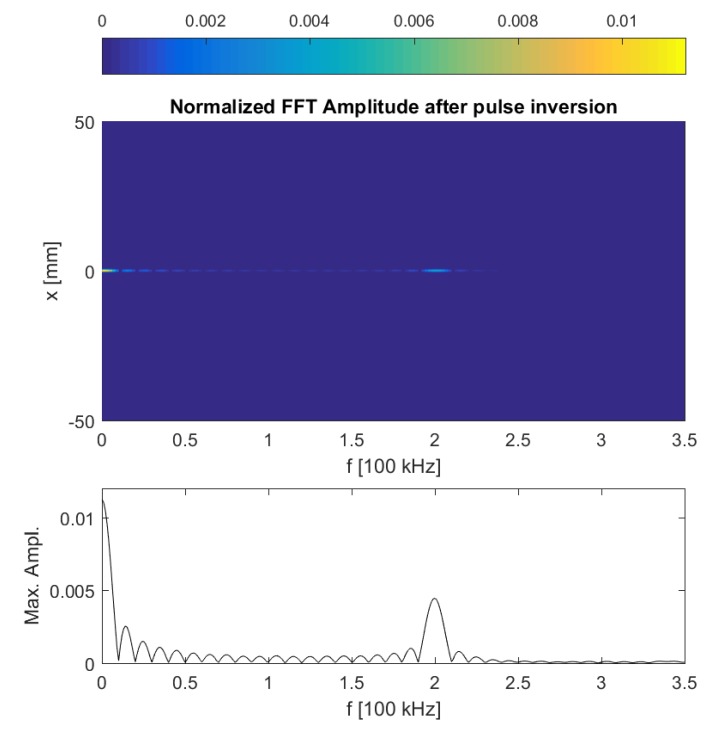
(Top) Frequency spectra obtained after applying the pulse inversion technique on the calculated normal displacement signals for a number of points on the top surface of the plate, with *x*-coordinates ranging from −50 mm to 50 mm. (Bottom) Normalized maximum FFT amplitude response measured along the top surface of the plate, after applying the pulse inversion technique. The figures clearly illustrate the generation of a second harmonic at the position of the crack (x=0).

Figure 2 shows a snapshot of the calculated *y*-component of the displacement field in the aluminum sample, clearly illustrating the presence of the excited $A_{0}$ guided Lamb wave. At the time instant shown in the figure, the guided wave passed the defect already and should therefore have started interacting with it. This interaction is not visible in the (linear) wave propagation itself, but will be evidenced by the generation of nonlinear features due to the clapping and frictional behavior of the crack. In Figure 3, the frequency spectra of the calculated normal displacements are shown for a number of points on the top surface of the sample, with *x*-coordinates ranging from −50 mm (i.e., leftmost position on the plate) to 50 mm. In this figure, however, no harmonics can be discerned. This is mainly due to the fact that the crack is too small to generate a large amount of nonlinearity, and hence, the (second order) nonlinear response is masked by a large linear response. In order to zoom in on the harmonic component, the pulse inversion technique can be applied [10]. Using this method, the nonlinear contribution of the received signals is enhanced by performing the experiment twice using two out-of-phase excitation signals with the same amplitude. This means that the polarity of excitation is changed: the positive parts (compression) in the first excitation signal will be negative (tension) in the second experiment. Since a crack behaves differently under tension than in compression, this will generate a nonlinear contribution that can be detected and extracted from the two responses by simply adding them together. This is illustrated in Figure 4, for the same conditions as in Figure 3. A second harmonic frequency is clearly generated at the position of the crack (x=0). The numerical results shown in Figure 3 and Figure 4 actually correspond to what can be obtained in real experiments using SLV and therefore illustrate the use of SLV for crack detection and localization.

### 2.2. Nonlinear Air-Coupled Emission

As mentioned before, harmonic components generated by clapping and frictional behavior of a crack will be radiated into the surrounding air. In order to determine the NACE radiation patterns at specific frequencies, a 2D spectral model for air is developed. The model geometry consists of a rectangular air domain of 10 cm by 4 cm, as illustrated in Figure 1. The left, right and top boundary of this domain are defined as matched boundaries in order to eliminate unwanted reflections coming from the edges of the computational region. At the bottom boundary, a normal acceleration is defined as a 1D input source. The accelerations used are directly related to the normal displacements calculated at the top surface of the plate using the previous model. Indeed, the obtained displacements are temporally Fourier transformed and filtered around a fixed response frequency (e.g., the fundamental frequency or its second harmonic) in order to be used in the acceleration boundary condition of the 2D spectral solution of air above the plate. This finally allows determining radiation patterns at specific frequencies.

The spectral problem is solved in COMSOL Multiphysics^®^, using the direct MUMPS solver. The air domain is meshed using quadratic, square elements. To reach convergence, COMSOL^®^ requires approximately six second-order mesh elements per wavelength. Since we like to have converging solutions for both the fundamental frequency and its second harmonic, the maximum element size is set to approximately 0.28 mm.

**Figure 5:**
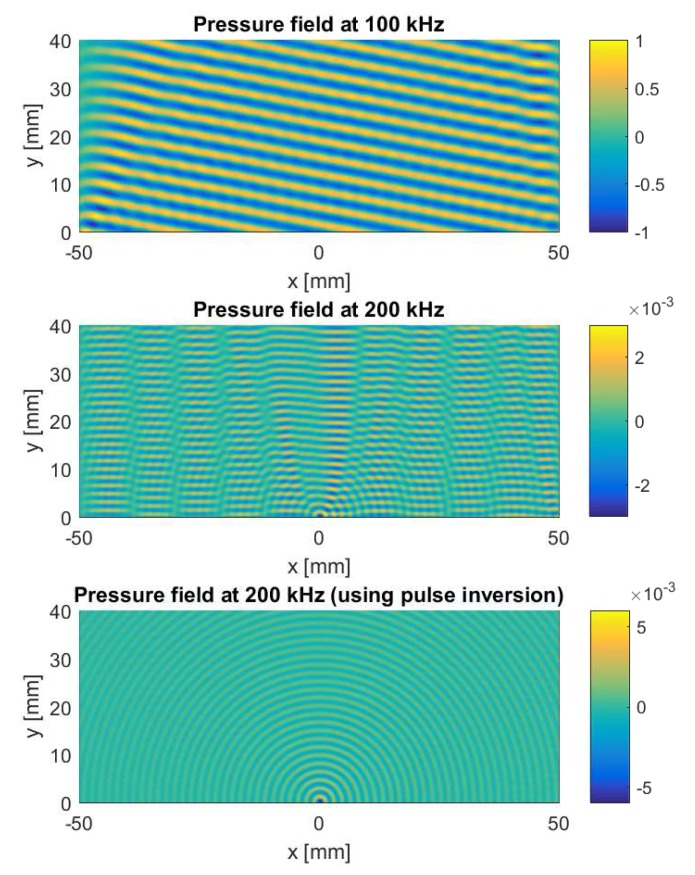
Radiation patterns in air above the aluminum plate with a near-surface crack. (Top) Fundamental frequency field showing no evidence of the presence of a crack. (Middle) Second harmonic field showing slight radiation of the harmonic into the air, starting from the crack position (x=0). (Bottom) Second harmonic field obtained after applying the pulse inversion technique. The crack clearly behaves as a source of nonlinear emission.

Figure 5 shows images of the calculated radiation patterns above the aluminum plate at the fundamental frequency of 100 kHz (top figure) and at its second harmonic (middle and bottom figure). The fundamental frequency field illustrates the typical radiation pattern observed in air surrounding a specimen in which a leaky Lamb wave is propagating. Once this Lamb wave encounters the near-surface crack, nonlinearities are generated, and the defect starts to behave as a localized source of nonlinear emission, radiating harmonics into the ambient air. This is already slightly visible in the middle figure. As before, the pulse inversion technique can be used to cancel out all linear contributions and highlight this nonlinear effect, as illustrated in the bottom figure.

## 3. Inverse Model: Defect Localization

In the previous section, the leaking of nonlinear waves from the defect sample into the ambient air was illustrated. In this section, we will extend the forward model by adding an array of ultrasonic air-coupled sensors that will be used to detect and extract the nonlinear airborne components, as already illustrated in Figure 1. The extracted signals will be used as an input in three different localization algorithms in order to find the exact location of the near-surface crack. The first method is a sum-and-delay technique, which actually consists of an explicit search over all possible defect locations. The second and third technique, on the other hand, allow computing the defect location directly.

The description of the localization algorithms in the following subsections is based on a number of conditions and assumptions. First, the considered sensor array consists of *N* sensors equidistantly spaced on a line. Such an array is referred to as a Uniform Linear Array (ULA). We also assume that the distance *D* from the sensor array to the sample is known and equals 3 cm. Moreover, since the considered localization algorithms are based on the phase information present in the ultrasonic signals, the distance *d* between the centers of each pair of sensors (i.e., pitch) should be smaller than half the wavelength of the impinging wave in order to avoid spatial aliasing [29]. In the present study, the second harmonic radiation field at 200 kHz needs to be detected. For this frequency, the wavelength in air is about 1.7 mm, so that in order to fulfill the half-wavelength criterion, the pitch *d* has to be smaller than approximately 0.8 mm. Currently, there are several examples described in the literature showing that it is possible to manufacture sensor arrays that meet this requirement [30,31,32,33]. Second, since the considered near-surface crack is very small (1 mm length), we may assume that, once excited, the defect will start to behave as a (secondary) point source. This was already observed in Figure 5. Third, due to the high attenuation in air at ultrasonic frequencies (i.e., the sensor array cannot be positioned too far from the sample) and the omni-directional NACE patterns in the case of small defects, the considered beamforming algorithms have to be developed for the near-field case. This case is more difficult to solve than the far-field problem and has therefore received less attention in the literature [34].

If we suppose that the time signal detected by sensor *k* (1≤k≤N) is denoted by $f_{k}\left(t\right)$, the vector containing all sensor signals is then given by:

(4) $$f\left(t\right)=\left(\begin{array}{c} f_{1}\left(t\right)\\ f_{2}\left(t\right)\\ \vdots \\ f_{N}\left(t\right) \end{array}\right)=\left(\begin{array}{c} f(t)\\ f(t-\tau_{2})\\ \vdots \\ f(t-\tau_{N}) \end{array}\right),$$

where $\tau_{k}$ is the time the signal detected by sensor *k* shifted with respect to the signal received by the first sensor. The Fourier transform F(ω) of the detected time signals f(t) is then defined as:

(5) $$F\left(\omega \right)=\left(\begin{array}{c} F_{1}\left(\omega \right)\\ F_{2}\left(\omega \right)\\ \vdots \\ F_{N}\left(\omega \right) \end{array}\right)=F\left(\omega \right)\left(\begin{array}{c} 1\\ e^{-i\omega \tau_{2}}\\ \vdots \\ e^{-i\omega \tau_{N}} \end{array}\right),$$

where $F_{k}\left(\omega \right)$ is the Fourier transform of signal $f_{k}\left(t\right)$ and F(ω) is the Fourier transform of signal f(t). The above formula can be considered for every possible frequency ω. However, we are only interested in the second harmonic behavior of the received signals (i.e., $\omega =2\omega_{0}$, with $\omega_{0}$ the fundamental angular frequency). In this case, F(ω) is a fixed complex value, which significantly eases the calculations. As can be seen, the received signals are all equal, apart from a phase shift due to the spatial separation of the sensors. This phase shift will be the basis for the three localization algorithms considered here.

### 3.1. Sum-And-Delay Approach

**Figure 6:**
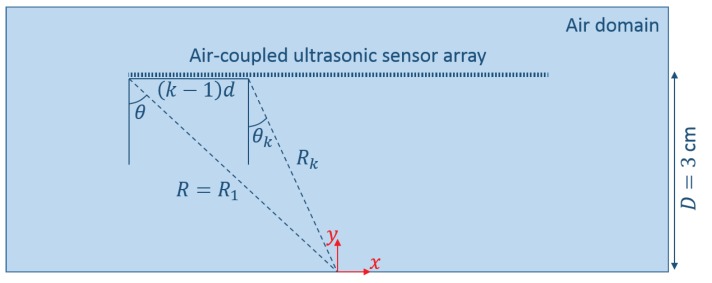
Representation of the near-field situation used in the beamforming algorithms.

Suppose $R=R_{1}$ is the distance from the leftmost sensor to the defect (located in the origin of the reference system), and θ is the angle formed by the line perpendicular to the sensor array and the line between the sensor and the defect, as illustrated in Figure 6. Using the first sensor as a reference, the distance $d_{1,k}$ between this sensor and sensor *k* is given by:

(6) $$d_{1,k}=\left(k-1\right)d.$$

Using the law of cosines, one easily finds that the distance $R_{k}$ between the defect and sensor *k* is given by:

(7) $$\begin{array}{c} R_{k}=\sqrt{R^{2}+{\left(k-1\right)}^{2}d^{2}-2R\left(k-1\right)d\sin\theta }. \end{array}$$

As a result, the time shift $\tau_{k}$ can be calculated as:

(8) $$\tau_{k}=\frac{R_{k}-R}{c},$$

where c=343.2 m/s is the velocity of sound in air, and R=D/cosθ.

The sum-and-delay method now works as follows. For every possible angle $\theta^{\prime }$ in the interval [−π/2,π/2], the time shifts $\tau_{k}^{\prime }$ are calculated according to Equation (8). Then, each signal $F_{k}\left(\omega \right)$ is shifted back in time according to this time shift, which in the frequency domain corresponds to a multiplication by $e^{i\omega \tau_{k}^{\prime }}$. Once this is done, the shifted signals are summed, resulting in the following signal:

(9) $$Y\left(\omega \right)=F\left(\omega \right)+F\left(\omega \right)e^{-i\omega \tau_{2}}e^{i\omega \tau_{2}^{\prime }}+\ldots +F\left(\omega \right)e^{-i\omega \tau_{N}}e^{i\omega \tau_{N}^{\prime }}=W^{*}F\left(\omega \right),$$

where W is the complex weight vector:

(10) $$W=\left(\begin{array}{c} 1\\ e^{-i\omega \tau_{2}^{\prime }}\\ \vdots \\ e^{-i\omega \tau_{N}^{\prime }} \end{array}\right),$$

and $W^{*}$ denotes the Hermitian transpose of W. Finally, the power of the received signal Y(ω) can be written as:

(11) $$P\left(\omega \right)=Y{\left(\omega \right)}^{2}=\left[W^{*}F\left(\omega \right)\right]{\left[W^{*}F\left(\omega \right)\right]}^{*}.$$

Repeating this procedure for every possible angle $\theta^{\prime }$, the correct angle θ is found as the angle that maximizes the power P(ω). The exact location of the defect ($x_{Crack}$) can then be derived from this angle θ and the fact that the sensor array is positioned a distance *D* from the plate:

(12) $$x_{Crack}=D\tan\theta +x_{1},$$

where $x_{1}$ is the *x*-coordinate of the first (leftmost) sensor in the array.

**Figure 7:**
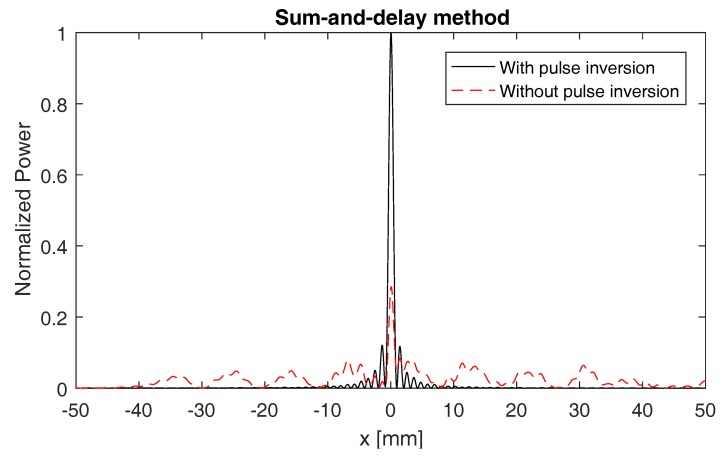
Normalized power *P* versus all possible defect locations. The power is calculated by applying the sum-and-delay approach on the second harmonic signals emitted by the defect. The sensor array used here contains 161 elements, ranging from x=−40 mm to x=40 mm and separated by a distance d=0.5 mm. The red, dashed line shows the result obtained without the use of pulse inversion (i.e., using the second harmonic signals from Figure 5, middle figure). The black, solid line shows the result obtained when using pulse inversion (i.e., using the second harmonic signals from Figure 5, bottom figure). In both cases, the exact location of the defect occurs at the maximum of the power function.

Figure 7 shows the power as a function of all possible defect locations when applying this method on the second harmonic signals emitted by the defect. The considered sensor array contains 161 elements, positioned from x=−40 mm to x=40 mm and separated by a distance d=0.5 mm. The red, dashed line shows the result obtained without the use of pulse inversion (i.e., using the second harmonic signals from Figure 5, middle figure). The black, solid line shows the result obtained when using pulse inversion (i.e., using the second harmonic signals from Figure 5, bottom figure). In both cases, the power function reaches its maximum at the exact location of the defect (x=0), with a higher signal-to-noise ratio in case pulse inversion was used.

**Figure 8:**
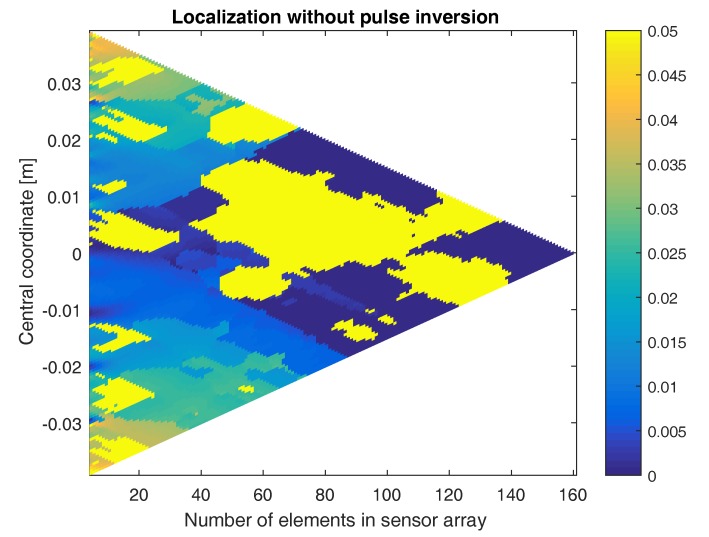
Color-coded plot of the difference between the exact defect location and the location obtained when applying the sum-and-delay approach using sensor arrays with varying numbers of elements and centered at different coordinates. The results were obtained using the second harmonic signals emitted by the defect (without using pulse inversion). Saturated yellow regions mean that the determined location is equal to or more than 5 cm away from the exact defect location.

**Figure 9:**
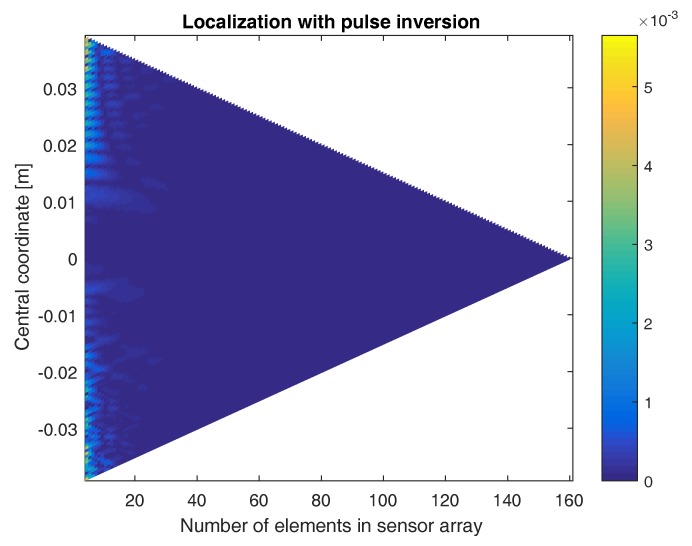
Color-coded plot of the difference between the exact defect location and the location obtained when applying the sum-and-delay approach using sensor arrays with varying numbers of elements and centered at different coordinates. The results were obtained using the second harmonic signals emitted by the defect, with the use of pulse inversion.

The above result already demonstrates the benefit of using the pulse inversion technique. It turns out that, in some cases, it is even impossible to find the defect location without the use of pulse inversion. In Figure 8, the location of the defect is determined using sensor arrays with a varying numbers of elements and centered at different coordinates. The figure shows a color-coded plot of the distance between the exact defect location and the location obtained when applying the sum-and-delay approach on the second harmonic signals emitted by the defect, without using pulse inversion. As can be seen, there are only a few sensor configurations where the defect location is well-determined. In some cases, the obtained location is even more than 5 cm away from the exact location (i.e., saturated yellow regions). If we repeat the same procedure using pulse inversion, Figure 9 shows that for all sensor array configurations, the defect location is well determined. The obtained location is never more than 6 mm away from the exact defect location. The higher the number of elements in the array, the better the localization. Furthermore, the closer the central coordinate of the array is positioned above the defect, the better the results. In what follows, we will only consider the pulse-inverted signals, as these will provide the best results.

### 3.2. Direct Linear Approach

The sum-and-delay approach applied on the pulse inverted signals turns out to be very accurate, mainly due to the fact that the algorithm is not making use of any approximation. This high accuracy, however, depends on the density of the parameter grid, which comes at the cost of higher computation times. In order to decrease the calculation time, it can be interesting to introduce some approximations, allowing one to determine the defect location in a direct and faster way.

For the considered problem of near-surface crack localization, the far-field assumption, assuming waves to be planar when arriving at the sensor array, cannot be used. However, if we consider only a small portion of the array, we can still use this assumption. For instance, splitting up the array into (possibly overlapping) sub-arrays of two sensors and using the far-field assumption R≫d, we can approximate Equation (7) by taking only the linear part of the Taylor expansion of $R_{k}$. This results in the equation:

(13) $$\begin{array}{c} R_{k}=R-\left(k-1\right)d\sin\theta . \end{array}$$

Using Equations (5) and (8), the relation between the signals of two successive sensors is then:

(14) $$F_{k+1}\left(\omega \right)=e^{i\frac{\omega }{c}d\sin\theta_{k}}F_{k}\left(\omega \right).$$

Hence, using the signals of two successive sensors, it is possible to determine the angle $\theta_{k}$ at which the wave impinges at the first of the two sensors. From this angle, we can determine a possible location $x_{Crack,k}$ of the defect using the following equation:

(15) $$x_{Crack,k}=D\tan\theta_{k}+x_{k},$$

where $x_{k}$ is the *x*-coordinate of the first of the two considered sensors. Repeating this procedure for each set of two successive sensors, we can determine the final location of the crack as the mean of all possible locations:

(16) $$x_{Crack}=\frac{1}{N-1}\sum_{k=1}^{N-1}x_{Crack,k}.$$

**Figure 10:**
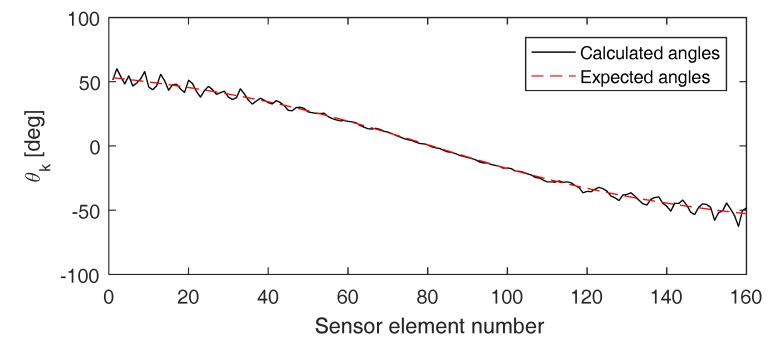
Angle $\theta_{k}$ versus sensor element number of the first of two successive sensors at which the wave impinges. The solid black line corresponds to the angles calculated using the direct linear approach. The dashed red line corresponds to the angles that are theoretically expected.

Figure 10 shows the angles $\theta_{k}$ found when applying this method on the (pulse inverted) signals detected by the sensor array used before (i.e., array of 161 elements separated by a distance of 0.5 mm). It can be seen that the calculated angles (solid black line) nicely correspond to the expected angles (dashed red line). From the calculated angles, we can determine the defect location $x_{Crack}=-0.19$ mm, which is very close to the exact defect location (x=0).

**Figure 11:**
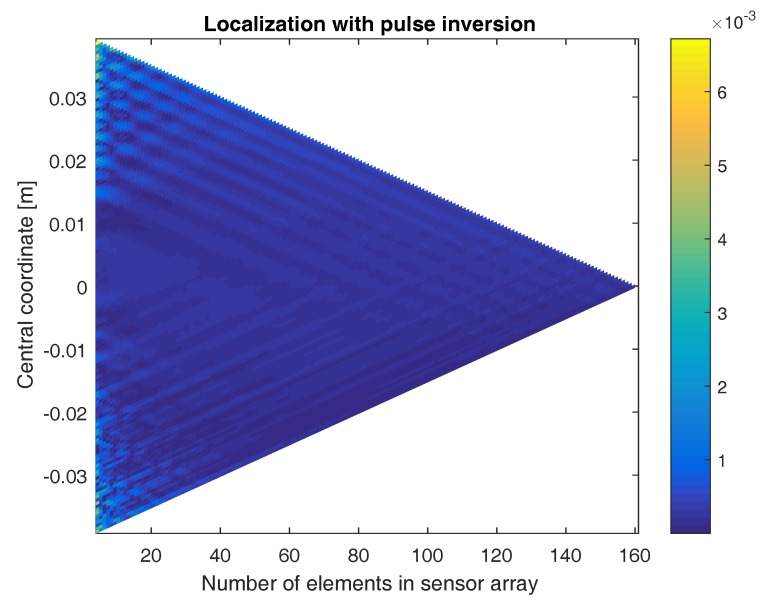
Color-coded plot of the difference between the exact defect location and the location obtained when applying the direct linear approach using sensor arrays with varying numbers of elements and centered at different coordinates. The results were obtained using the second harmonic signals emitted by the defect, with the use of pulse inversion.

In Figure 11, the location of the defect is again determined using sensor arrays with a varying numbers of elements and centered at different coordinates. The figure shows a color-coded plot of the distance between the exact defect location and the location obtained when applying the direct linear approach on the second harmonic signals emitted by the defect and when using pulse inversion. Similar to the sum-and-delay approach, good localization results are obtained for all sensor array configurations. As before, the best results are obtained for a high number of elements in the array and for arrays centered above the defect. Even though this approach uses a linear approximation, the accuracy of the results is in the same order of magnitude as what was obtained using the sum-and-delay approach. Note, however, that the accuracy of the sum-and-delay method can be easily increased by working on a denser grid in the search space.

### 3.3. Direct Quadratic Approach

The planar wave assumption on which the direct linear approach is based can not always be used. For instance, when the sensor array is positioned too close to the sample or when larger sub-arrays are considered (i.e., arrays of more than two elements), the inherent curvature of the wavefronts impinging on the array is no longer negligible. In these situations, we can include the next term in the Taylor expansion of $R_{k}$ (Equation (7)), such that the waves in the proximity of the ultrasonic sensor array are considered to be quadratic. This results in the following equation:

(17) $$R_{k}=R-\left(k-1\right)d\sin\theta +\frac{{\left(k-1\right)}^{2}d^{2}\cos^{2}\theta }{2R},$$

which is known as the Fresnel approximation [34]. Using Equations (5) and (8), the signals received by four successive sensors can be written as follows:

(18) $$\begin{array}{c} F_{k}\left(\omega \right)=F\left(\omega \right)e^{i\omega \left[\left(k-1\right)\alpha -{\left(k-1\right)}^{2}\beta \right]}, \end{array}$$

(19) $$\begin{array}{c} F_{k+1}\left(\omega \right)=F\left(\omega \right)e^{i\omega \left[k\alpha -k^{2}\beta \right]}, \end{array}$$

(20) $$\begin{array}{c} F_{k+2}\left(\omega \right)=F\left(\omega \right)e^{i\omega \left[\left(k+1\right)\alpha -{\left(k+1\right)}^{2}\beta \right]}, \end{array}$$

(21) $$\begin{array}{c} F_{k+3}\left(\omega \right)=F\left(\omega \right)e^{i\omega \left[\left(k+2\right)\alpha -{\left(k+2\right)}^{2}\beta \right]}, \end{array}$$

where:

(22) $$\alpha =\frac{d\sin\theta_{k}}{c},$$

and:

(23) $$\beta =\frac{d^{2}\cos^{2}\theta_{k}}{2cR},$$

with $\theta_{k}$ the angle at which the wavefront impinges at the first of the four considered sensors. A simple computation shows that we can eliminate α as follows:

(24) $$\frac{F_{k}\left(\omega \right)F_{k+3}\left(\omega \right)}{F_{k+1}\left(\omega \right)F_{k+2}\left(\omega \right)}=e^{-i\omega 4\beta }.$$

Hence, using the signals of four successive sensors, it is possible to determine the parameter β. Once β is known, we can determine new signals $G_{k}\left(\omega \right)$ till $G_{k+3}\left(\omega \right)$ as follows:

(25) $$\begin{array}{c} G_{k}\left(\omega \right)=F_{k}\left(\omega \right)e^{i\omega {\left(k-1\right)}^{2}\beta }, \end{array}$$

(26) $$\begin{array}{c} G_{k+1}\left(\omega \right)=F_{k+1}\left(\omega \right)e^{i\omega k^{2}\beta }, \end{array}$$

(27) $$\begin{array}{c} G_{k+2}\left(\omega \right)=F_{k+2}\left(\omega \right)e^{i\omega {\left(k+1\right)}^{2}\beta }, \end{array}$$

(28) $$\begin{array}{c} G_{k+3}\left(\omega \right)=F_{k+3}\left(\omega \right)e^{i\omega {\left(k+2\right)}^{2}\beta }, \end{array}$$

from which it follows that:

(29) $$\left(\begin{array}{c} G_{k+1}\left(\omega \right)\\ G_{k+2}\left(\omega \right)\\ G_{k+3}\left(\omega \right) \end{array}\right)=e^{i\omega \alpha }\left(\begin{array}{c} G_{k}\left(\omega \right)\\ G_{k+1}\left(\omega \right)\\ G_{k+2}\left(\omega \right) \end{array}\right).$$

Solving this equation allows finding α, from which we can deduce $\theta_{k}$ using Equation (22). Similar to the direct linear approach, the angle $\theta_{k}$ can be used to determine a possible location $x_{Crack,k}$ of the defect using the following equation:

(30) $$x_{Crack,k}=D\tan\theta_{k}+x_{k},$$

where $x_{k}$ is now the *x*-coordinate of the first of the four considered sensors. Repeating this procedure for each set of four successive sensors, we can again determine the final location of the defect as the mean of all possible locations:

(31) $$x_{Crack}=\frac{1}{N-3}\sum_{k=1}^{N-3}x_{Crack,k}.$$

**Figure 12:**
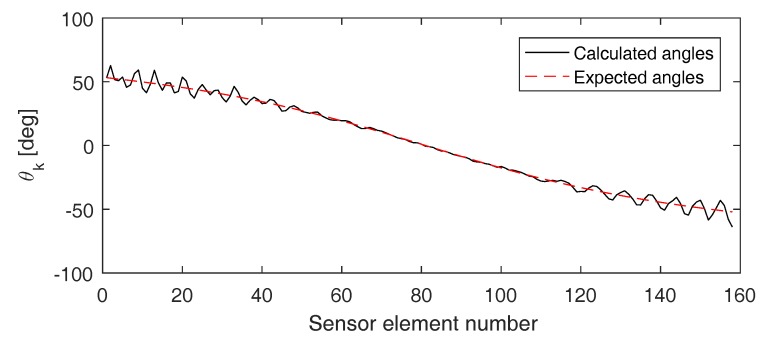
Angle $\theta_{k}$ versus sensor element number of the first of four successive sensors at which the wave impinges. The solid black line corresponds to the angles calculated using the direct quadratic approach. The dashed red line corresponds to the angles that are theoretically expected.

Figure 12 shows the angles $\theta_{k}$ found when applying this method on the (pulse inverted) signals detected by the sensor array used before (i.e., array of 161 elements separated by a distance of 0.5 mm). It can again be seen that the calculated angles (solid black line) nicely correspond to the expected theoretical values (dashed red line). From the calculated angles, we can determine the defect location $x_{Crack}=-0.06$ mm, which is very close to the exact defect location (x=0).

**Figure 13:**
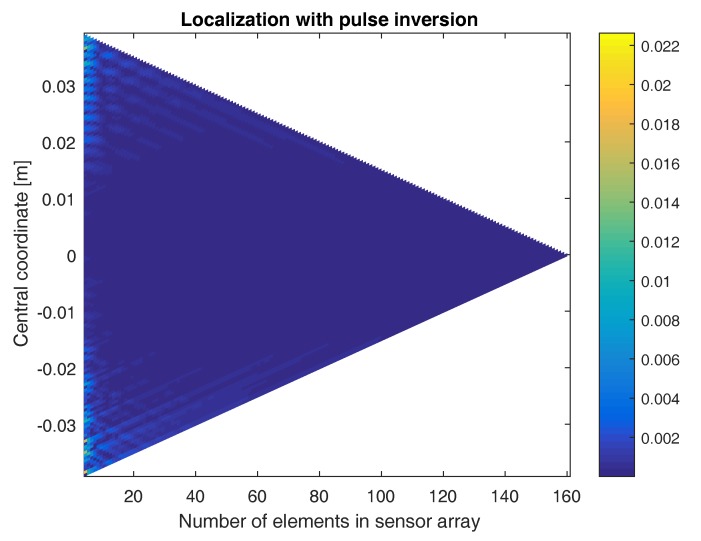
Color-coded plot of the difference between the exact defect location and the location obtained when applying the direct quadratic approach using sensor arrays with varying numbers of elements and centered at different coordinates. The results were obtained using the second harmonic signals emitted by the defect, with the use of pulse inversion.

In Figure 13, the location of the defect is again determined using sensor arrays with a varying numbers of elements and centered at different coordinates. The figure shows a color-coded plot of the distance between the exact defect location and the location obtained when applying the direct quadratic approach on the second harmonic signals emitted by the defect and when using pulse inversion. As with the two previous approaches, qualitatively good results are obtained for all sensor array configurations. The more sensor elements in the array and the closer the central coordinate of the array is above the defect, the better the results. The accuracy of the quadratic approach seems to be less good than what was obtained using the linear approach, especially when considering small sensor arrays. This is probably due to the fact that at a distance of D=3 cm (i.e., the distance from the considered sensor arrays to the aluminum sample), the waves impinging at the sensor array can already be assumed more or less planar.

**Figure 14:**
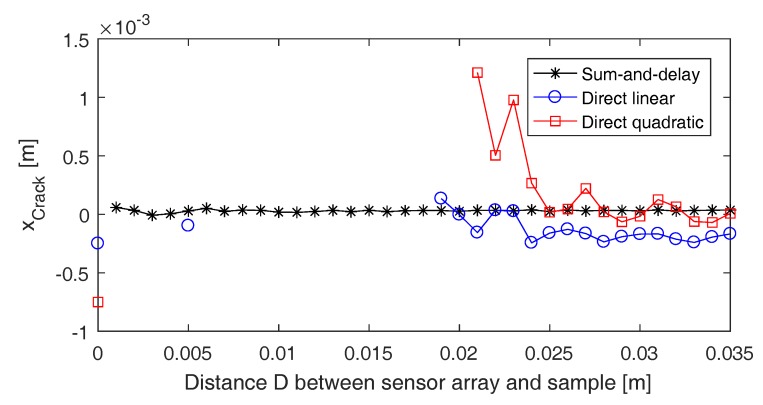
Calculated defect location $x_{Crack}$ versus the distance *D* from the sensor array to the test surface. The sensor array used contains 161 elements, ranging from x=−40 mm to x=40 mm and separated by a distance d=0.5 mm. Three different approaches were used to determine the crack location: the sum-and-delay approach (crosses), the direct linear approach (circles) and the direct quadratic approach (squares). Only those crack locations that are closer than 1 cm to the exact location of the defect (i.e., x=0) are shown in the graph.

As mentioned earlier, both the linear and quadratic approach are expected to be less accurate than the sum-and-delay approach because they are based on a number of assumptions. This will particularly be the case when the sensor array is positioned closer to the test sample (i.e., in the near-field of the defect source), where the planar or quadratic approximation of the impinging wavefronts is not valid anymore. To illustrate this, we determined the crack location $x_{Crack}$ applying the three different approaches on the (pulse inverted) signals detected by the full sensor array (i.e., array of 161 elements separated by a distance of 0.5 mm) while changing the distance *D* from the sensor array to the test surface. The results are shown in Figure 14. For the sake of clarity, only the calculated locations that are closer than 1 cm to the exact crack location are plotted (the others can be considered as bad or wrong localizations). As expected, the sum-and-delay approach always results in good localization of the defect. The direct linear and quadratic approaches, on the other hand, are only capable of determining a decent defect location when the sensor array is more than approximately 2 cm away from the test surface. The larger the distance *D* from the sensor array to the test sample, the higher the accuracy of the direct approaches. This is something that has to be taken into account when using these approaches in real experiments.

## 4. Conclusions

In the current paper, a numerical study was performed in order to test the capability of an air-coupled ultrasonic sensor array to localize near-surface cracks in plate-like samples. Two models were developed: a forward model, used to simulate the generation and emission of nonlinearities, and an inverse model that uses localization algorithms to find the exact location of a defect.

The forward model is implemented in the finite element-based software package COMSOL Multiphysics^®^ and consists of two parts. The first part is a 2D time domain model that allows the simulation of (nonlinear) Lamb wave propagation in cracked samples and takes into account the clapping and frictional behavior of the crack, resulting in the generation of nonlinear features, such as the presence of harmonics. The second part is a 2D spectral model in air that allows determining NACE radiation patterns resulting from harmonic components radiating from the defect sample into the surrounding air. The forward model was illustrated for guided Lamb wave propagation in an aluminum plate with a horizontally-oriented near-surface crack of 1 mm in length and positioned at a depth of 0.2 mm.

For the inverse model, an array of ultrasonic air-coupled sensors was used to detect the second harmonic nonlinear airborne components. Using these signals, the location of the defect could be determined by means of three different near-field localization algorithms: a sum-and-delay approach, a direct linear approach and a direct quadratic approach. All three algorithms allow good defect localization, especially when using the sensor signals obtained after applying the pulse inversion technique. The best results were obtained for large sensor arrays of which the center was positioned above the defect. In practice, the sum-and-delay approach will be the most accurate one, as this method is not making use of approximations. As a drawback, the sum-and-delay method is based on an explicit search over the parameter space, which requires high computation times to reach a desired level of accuracy. The direct approaches, on the other hand, are based on a number of approximations, making them less accurate, but much faster to solve. A combination of both methods, where the direct approaches are used to obtain a quick guess of the defect location, followed by the sum-and-delay approach in which the spatial parameters are swept around that particular location, may possibly overcome the shortcomings of both methods.

In the future, we would like to study alternative approaches to improve the quality of single defect localization. One approach is to repeat the localization procedure for multiple frequency components in the nonlinear spectrum and to fuse the obtained results. Another approach would be to use broadband signals. Since the arrays considered in this paper are uniform linear arrays of which the distance between the elements is quite small, we also would like to check whether all sensors are really required to obtain a good estimate of the defect position. The obvious advantage of using fewer sensors is that the electronics and software for the control of the ultrasonic sensor array system would become much simpler. Furthermore, it would improve the robustness of the system, as possibly malfunctioning sensors can be left out of the system. Finally, working with fewer sensors will efficiently reduce the application costs in the potential final design of an ultrasonic air-coupled sensor system for incipient damage localization.

## Abbreviations

The following abbreviations are used in this manuscript:

DOA	Direction Of Arrival
MMD	Method of Memory Diagrams
NACE	Nonlinear Air-Coupled Emission
NDT&E	Non-Destructive Testing and Evaluation
NEWS	Nonlinear Elastic Wave Spectroscopy
SAT	Sparse Array Tomography
SLV	Scanning Laser Vibrometry
TR	Time Reversal
ULA	Uniform Linear Array
